# ObTiMA

**DOI:** 10.1186/2043-9113-5-S1-S3

**Published:** 2015-05-22

**Authors:** Holger Stenzhorn

**Affiliations:** 1 Saarland University Medical Center, 66424 Homburg, Germany

## Characterisation

Ontology-based Clinical Trial Management Application, clinical trial data management.

## Tool description

ObTiMA is an ontology-based clinical trial data management system [1] to support clinicians in designing and conducting clinical trials. The Trial Builder in which all trial aspects can be specified facilitates the design phase; it is therefore possible to define the outline as well as metadata of a trial in a master protocol including trial goals or administrative data.

**Figure 1:**
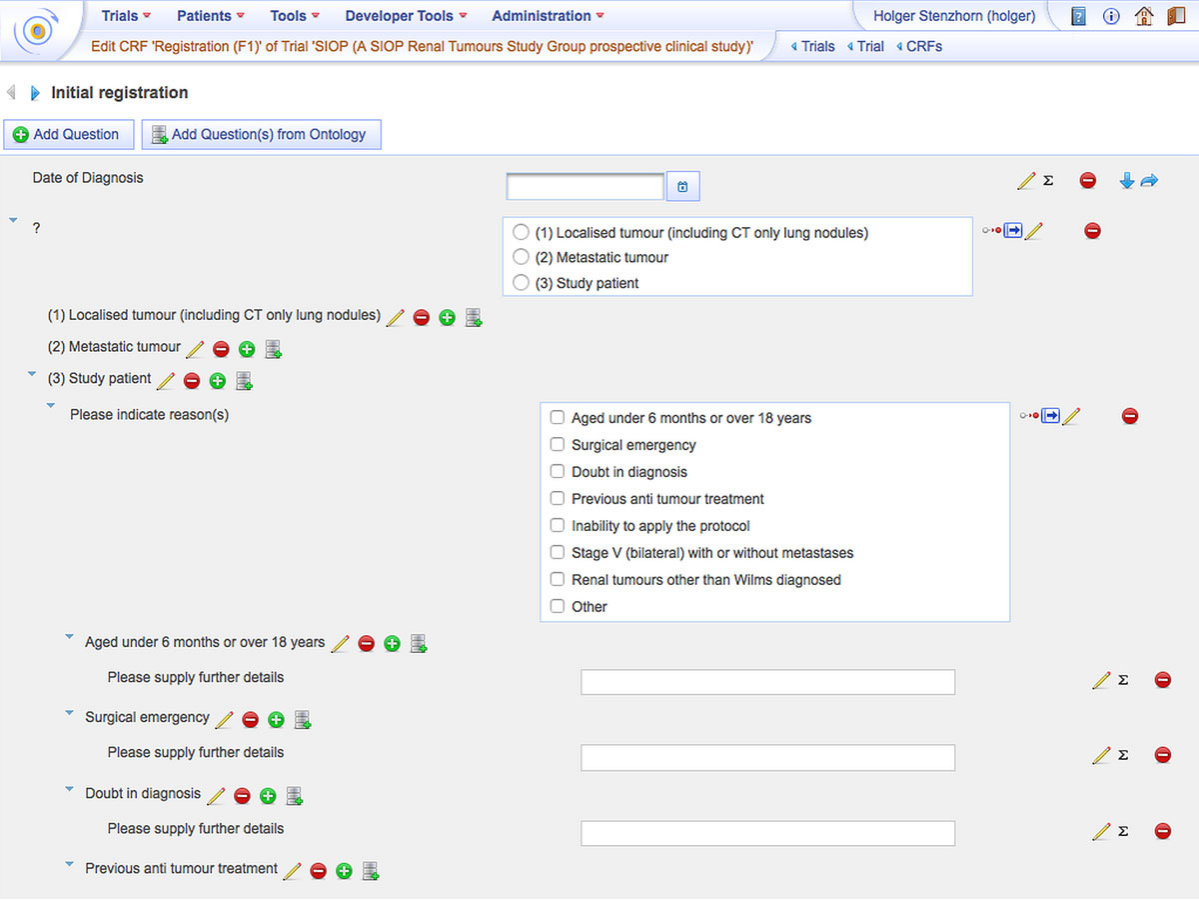
ObTiMA user interface. Display of creating a CRF to register a patient in the SIOP trial.

The ontology-based CRF (case report form) creation module of the Trial Builder is one of the systems core functionalities (Figure 1). A graphical user interface allows defining content, navigation, and layout of CRFs to capture all patient data during a trial. The resulting descriptions are based on ontological concepts for each CRF item along with metadata, such as data type and measurement unit. The user interface makes the underlying ontological aspects transparent to shield the user from complex logical data representations. For example, if an item has been created based on a concept, its attributes, like label, data type or answer possibilities, are determined automatically (but they can also be adopted manually if necessary).

Since many trials collect similar types of data, it is useful to store CRF forms in a template repository for reuse. When setting up a trial these templates can be reused and adapted to the new trial's needs.

ObTiMA's second core functionality is the management of patient data. The setup of this modul is done automatically based on the items defined in the trial builder and an easy to use interface is provided to enter patient data into CRFs. A protection framework that de-identifies all patient-related data and role-based access control that enables fine-grained control over access ensure the security of all collected data. Furthermore, all system access and changes to data are permanently logged via an audit trial. In summary, the ObTiMA system has been developed in a modular way allowing the straightforward extension of its core functionalities. Currently modules to integrate DICOM images with collected CRF data and a module to facilitate the reporting of Serious Adverse Events are being developed.

## Status of development

The prototype is being validated for GCP compliance.

## Users

SIOP D-A-CH.

## Link

http://obtima.org
